# A derivation of Maxwell's equations using the Heaviside notation

**DOI:** 10.1098/rsta.2017.0447

**Published:** 2018-10-29

**Authors:** Damian P. Hampshire

**Affiliations:** Superconductivity Group, Centre for Materials Physics, Department of Physics, University of Durham, South Road, Durham DH1 3LE, UK

**Keywords:** Heaviside, Maxwell's equations, Coulomb's Law, Ampere's Law, electromagnetism, charge conservation

## Abstract

Maxwell's four differential equations describing electromagnetism are among the most famous equations in science. Feynman said that they provide four of the seven fundamental laws of classical physics. In this paper, we derive Maxwell's equations using a well-established approach for deriving time-dependent differential equations from static laws. The derivation uses the standard Heaviside notation. It assumes conservation of charge and that Coulomb's law of electrostatics and Ampere's law of magnetostatics are both correct as a function of time when they are limited to describing a local system. It is analogous to deriving the differential equation of motion for sound, assuming conservation of mass, Newton's second law of motion and that Hooke's static law of elasticity holds for a system in local equilibrium. This work demonstrates that it is the conservation of charge that couples time-varying ***E***-fields and ***B***-fields and that Faraday's Law can be derived without any relativistic assumptions about Lorentz invariance. It also widens the choice of axioms, or starting points, for understanding electromagnetism.

This article is part of the theme issue ‘Celebrating 125 years of Oliver Heaviside's ‘Electromagnetic Theory’’.

## 1. Introduction

This research paper is written in the celebration of 125 years of Oliver Heaviside's work *Electromagnetic* *theory* [1]. Heaviside was broadly self-taught, an eccentric and a fabulous electrical engineer. He very probably first read Maxwell's great treatise on electricity and magnetism [2] while he was in the library of the Literary and Philosophical Society of Newcastle upon Tyne, just up the road from Durham [3]. He called Maxwell ‘heaven-sent’ and Faraday ‘the prince of experimentalists' [1]. Heaviside restructured Maxwell's original 20 equations to be the four equations that we now recognize as Maxwell's equations. In every high school, good physics students can write down Newton's laws. In every university, they can write down Maxwell's equations in the mathematical form developed by Heaviside.

Axioms in mathematics play a central pedagogical role in learning and understanding this discipline. In 300 BC, Euclid wrote *Elements*, his seminal text about geometrical mathematics [4]. It included his 10 axioms and the proofs of more than 400 propositions or theorems. It has provided the template for the logical approach that students have used over the following two-and-a-half millennia. Students demonstrate their knowledge and skill by using the axioms as starting points and derive all the consequences that follow. Axioms in science are usually taken to be generally true, or at least very widely true, and are distinguished from those more limited statements or equations that can be derived from the axioms and then used to describe a particular system or to provide results for an examination. Hence, we expect the professional mathematical and scientific communities to specify the axioms of their disciplines clearly and mark the development of new knowledge by changes in axioms. There is also the expectation that the most useful axioms, as the Greek word axioma (self-evident truth or starting point) suggests, cannot be derived from other equations or laws.

Probably, the most famous physics textbook of modern times is the three-volume textbook *The Feynman lectures on physics*. In it, Feynman says ‘we can understand the complete realm of classical physics’ from just seven equations [5]. The first three equations describe forces: Newton's law of motion, Newton's law of gravity and the force law for a charged particle moving in a magnetic and electric field. The remaining four are Maxwell's differential equations. Students of electromagnetism are introduced to Maxwell's equations and taught that they are generally true, not least because of the overwhelming body of experimental data that validates them. Not only do they describe the ***E***-fields and ***B***-fields from charges and currents in vacuum but by considering the charges and currents produced in materials, they describe the fields produced by all the important technologically useful materials and an enormous range of physical phenomena in the world around us. They also include a prohibition on the creation of net charge that is consistent with all experimentation to date.

Maxwell's original work used a heuristic approach to derive 20 scalar equations that describe electromagnetism and was first to demonstrate that light is a transverse electromagnetic wave. The equations have a form that follows Newton and emphasize the electromotive force produced by electric and magnetic fields, as shown in table 1. Heaviside, who was first employed as an engineer in the British Post Office telegraph system in Newcastle upon Tyne [3], took the equations, eliminated the vector and scalar potentials and developed the differential vector calculus notation necessary to write them down in the form that we currently use [8]. Heaviside's form gives the ***E***- and ***B***-fields an importance beyond the forces they can produce and opens the way to describe wave and energy propagation more directly.

**Table 1.** Maxwell's 20 (scalar) equations in modern form, labelled with his original lettering notation (A)–(H) [6]. The first 18 of his equations, (A)–(F), are given here as six vector equations using Heaviside's curl notation. There are also two scalar equations, (G) and (H). We have avoided Maxwell's use of ‘electromotive force’ and ‘actual electromotive force’ and taken 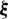 as the electromotive force per unit charge. Also, 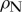 is the resistivity, 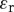 is relative permittivity and 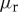 is relative permeability. Standard symbols have their usual meanings [7]. Equations (A)–(D) and (G) include what are now known as Maxwell's four equations together with the expression for the Lorentz force. The subscript ‘free’ that is used now for charge densities and current densities that can travel over macroscopic distances, was called ‘true conduction’ by Maxwell. He also considered the magnetic vector potential ***A*** in terms of the electromagnetic momentum per unit charge.

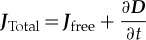	(A)	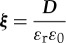	(E)
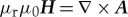	(B)	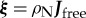	(F)
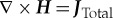	(C)	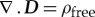	(G)
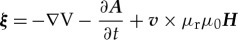	(D)	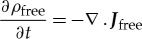	(H)

The historical development of electromagnetism has influenced its modern-day teaching. Undergraduate textbooks derive the electrostatic and magnetostatic differential equations mathematically from Coulomb's Law and Ampère's Law. However, to arrive at Maxwell's time-dependent equations, students follow the heuristic approach. Most science students are then taught relativity without understanding properly the axioms of classical electromagnetism. This is pedagogically unsound because if we do not make explicit the axioms of classical electromagnetism, in the (albeit unlikely) event that there are new experiments that are not consistent with current understanding, we undermine our students' ability to identify which axioms can be retained and which ones should be discarded. For example, many students think that Faraday's Law is axiomatic or that the postulates of relativity are required to derive Faraday's Law. In this paper, we show that Faraday's Law can be derived without using any of the assumptions from Einstein's theory of relativity. Indeed, the derivations here beg the question as to whether Coulomb's Law, Ampère's Law and Faraday's Law should all have the status of laws at all, given that we can derive Faraday's Law from the other two.

The next section of this paper discusses the process by which static laws can be used to derive time-dependent differential equations. As an exemplar, it considers the textbook use of Hooke's static law of elasticity to derive the time-dependent differential equation that describes the propagation of sound. Section 3 uses a similar approach to derive Maxwell's equations. We apply the vector calculus approach developed by Heaviside [9] to derive all four of Maxwell's equations. Finally, we speculate about possible sources of experimental evidence for the breakdown of Maxwell's equations.

## 2. Deriving time-dependent differential equations from static laws

Scientists are well versed in using static laws to derive time-dependent partial differential equations. To derive the time-dependent differential equation for the propagation of sound, we start with Hooke's static law of elasticity, which when used to describe static equilibrium in a gas, can be written
2.1
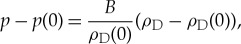

where *B* is the bulk modulus, 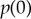 and 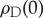 are the initial pressure and density of the gas under test, *p* is the applied pressure and 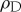 the resultant density. Hooke's static law is then rewritten as
2.2
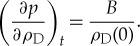


Equations (2.1) and (2.2) are quite different types of equations. Equation (2.1) relates how a change in the external pressure applied to a uniform and static gas changes the density throughout the entire gaseous system. Equation (2.2) is a differential equation that describes how a differential pressure across an infinitesimal volume causes a differential change in density. We note that equation (2.2) is derived by considering an element in which the cause (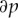) and the effect (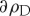) are infinitesimally close together (i.e. local). We describe the gas as being in local equilibrium, so that even though the pressure and density can vary as a function of space and time, every point throughout the system has local values of 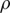 and *p* related by Hooke's Law. Local equilibrium also ensures that the differential of pressure with respect to space or time is related to an equivalent differential for density, for example, by an equation of the form
2.3
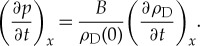


However, it is important to note that strictly, it is *not possible* to derive either equation (2.2) or (2.3) from (2.1) using mathematics alone. In (2.1), 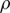 and 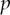 do not include the variables *x* and *t* (i.e. space and time), whereas in (2.2) and (2.3), they are functions of *x* and *t*. Textbooks do not usually emphasize that we have used our physical intuition and followed Occam's razor [10], so that among the many possible dependencies that include the time dependence for *p* and 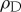, we have selected the one with the fewest additional assumptions.

To derive the equation that describes the propagation of sound, we then use Newton's second law of motion in the form
2.4
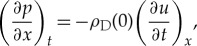

where 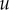 is the velocity and *t* is the time. Using the identity 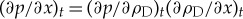 and equation (2.2), Newton's Law gives
2.5
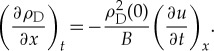


We then use the conservation of mass:
2.6
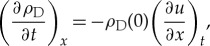

and partially differentiating (2.5) with respect to *t* and partially differentiating (2.6) with respect to *x* and allowing changes in the order of differentiation, we find
2.7
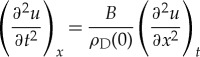


From (2.7), the velocity of sound *v* is given by 
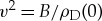
. The extension of Hooke's static law to the time domain allows us to describe a whole new range of phenomena associated with pressure waves (e.g. sound). However, this derivation also serves as a useful reminder of the limitations with this approach. In practice, the propagation of sound in a gas does not strictly obey (2.7), because propagation depends on how the temperature changes while the pressure is changing. Experimental results show that new physics, not found in the static measurements, is relevant in time-dependent systems (i.e. the rate of heat flow). There are other examples of systems in physics, where we start with a static law and can derive time-dependent differential equations some of which to first order do not require additional terms (e.g. in deriving dispersion relations such as the classical derivation for magnons using the Heisenberg spin Hamiltonian) and other examples where additional frictional terms are added (e.g. in describing energy loss such as in dispersive resonant polarisation in dielectrics). Hence, we emphasize that the validation of any time-dependent equations is ultimately an issue for experimentation. In this paper, we follow the simple approach described in equations (2.1)–(2.7). We postulate that if Coulomb's law of electrostatics and Ampère's law of magnetostatics are limited to describe what could be called ‘local equilibrium’—a local point of observation with local charges and current densities (i.e. local cause and effect), and written in the most simple time-dependent form (invoking Occam's razor), then the derivatives of these laws, the differential equations with respect to time and space, hold throughout the whole system. The philosophy of this approach looks to make the system sufficiently general that it includes all the components necessary to provide general differential equations.

## 3. Derivations of Maxwell's four equations

### (a) The divergence of ***E***

**Figure 1.**
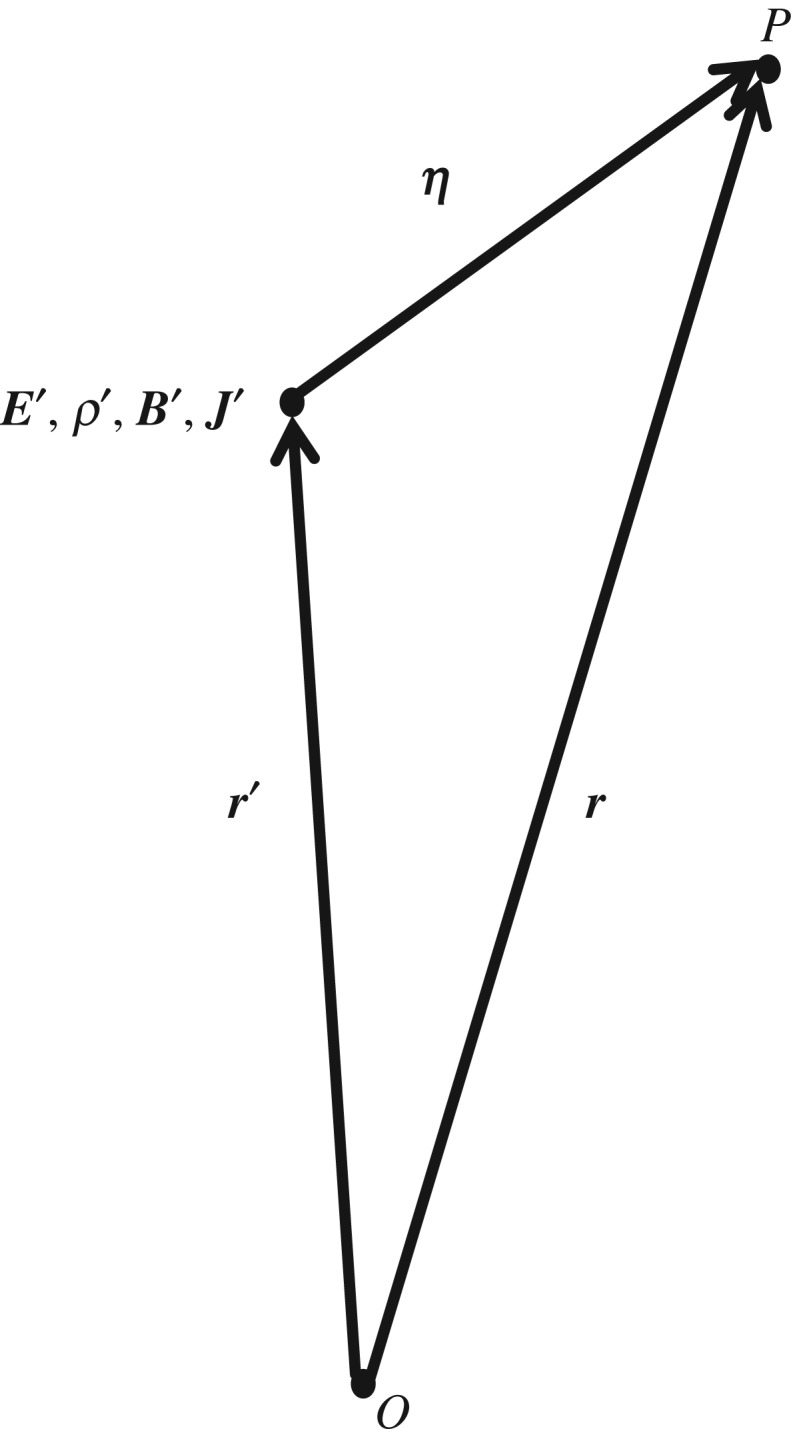
A coordinate system in which charge densities and current densities are observed. *O* is the origin. *P* is the point of observation. Charge densities and current densities are displaced from the origin at points 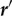. The vector separation between the charge density 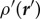 or current density 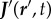 and the observation point is given by 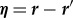. The electric and magnetic fields at the primed locations are 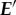 and 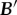
_,_ respectively. For the special case of the electric field, charge density, magnetic field and current density at the point of observation, we use unprimed values 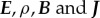
_,_ respectively.

The most simple generalization of Coulomb's law of electrostatics, to a time-dependent form where the point of observation and the charges present are local, is
3.1
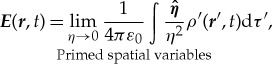

where as shown in figure 1, the electric field, 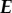, at a point of observation *P* located at a point 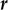 (*x*, *y*, *z*) and time *t*, is produced by the charge densities 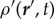 located at the primed points 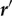 (*x′*, *y′*, *z′*) at the same time *t*. By definition, 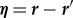
and 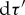 denotes integrating over the primed spatial variables of the charge densities while the unprimed spatial variables remain constant. Neither the spatial variables *x*, *y* and *z*, nor *x′*, *y′* and *z′* are functions of time. 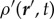 is the charge density at position 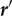 and time *t*. ***E*** is a function of the unprimed spatial variables *x*, *y* and *z* as well as time *t*. We assume that all the charge densities are local—very close to the point of observation. Hence, the partial time derivative of the ***E***-field at the point of observation is
3.2
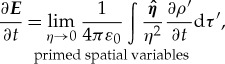

where 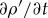 is calculated at time *t*. As is standard convention, partial derivatives with respect to time are calculated assuming all spatial variables (i.e. primed and unprimed) are held constant. We state the standard definition of the del operator ∇:
3.3
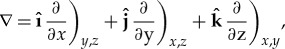

and note that for this operator, in addition to the unprimed spatial variables that are explicitly shown to be held constant, for each of the partial derivatives, the variable *t* and the primed variables *x′*, *y′* and *z′* are also held constant. Equations (3.1) and (3.3) lead to
3.4
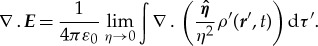

Using the identities 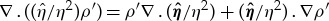
and 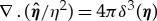 and noting that 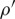 only depends on primed variables and the time *t*, we obtain one of Maxwell's equations,
3.5
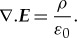


Equation (3.5) has the same form and uses similar mathematical identities to those used to derive the standard result from electrostatics. However, in this paper, we derive it from local time-dependent equations and then assume it is one of the underlying or fundamental differential equations that is correct at all points in space and time. Maxwell's equations have no agreed order. We call it Maxwell's first equation.

### (b) The divergence of ***B***

We use Ampère's law of magnetostatics and again invoke Occam's razor to postulate that the local time-dependent 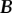-field at time *t* is
3.6
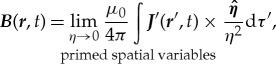


where 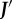 is only a function of the primed spatial variables and the time is *t*. Again we assume that equation (3.6) is only valid for a system where all the current densities are local to the point of observation. We can also write
3.7
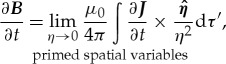

where 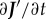 is calculated at *t*. To improve brevity, we will omit including 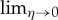 in the integral equations and the limits of the integration in this paper hereafter. Using a general vector field identity written in the form 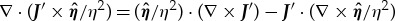 [7], given 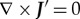 (because ∇ is not primed but 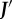 is primed), the divergence of (3.6) leads to
3.8
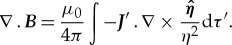


Using the vector field identity 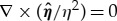 leads to the second of Maxwell's equations:
3.9
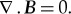


This equation also has the same form and uses similar mathematical identities to those used to derive the standard result from magnetostatics. However, as noted above, we have derived it from local time-dependent equations and assume it is correct at all points in space and time.

### (c) The curl of ***B***

#### (i) Maxwell's displacement current density

Textbooks [11] show, by taking the curl of both sides of Ampère's magnetostatic law, that
3.10
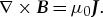


Maxwell realized that equation (3.10) cannot be generally true as a function of time, given the vector field identity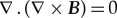. By invoking the continuity of charge equation given by 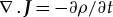 and considering the partial time derivative of (3.5), he added his famous displacement current density term 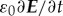 to equation (3.10) to give the third of his equations:
3.11
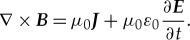


**Figure 2.**
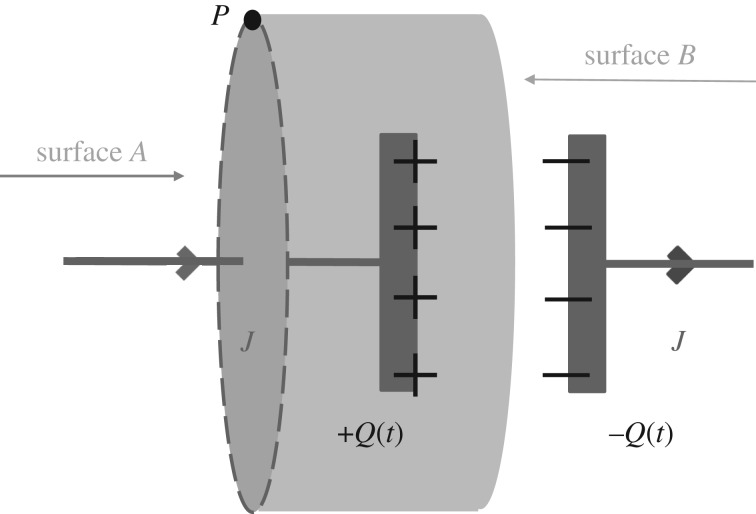
Straight wires carrying a constant current density 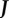 and charging two capacitor plates. Surface *A* is the flat circular surface bounded by the dotted ring path on which the point *P* is located and through which the current passes. Surface *B* is bounded by the same dotted ring path but passes through the capacitor plates so no current passes through it. The magnitude of the charge on each plate increases with time *t* and has magnitude *Q* (*t*).


Another approach used to justify the generalization from the magnetostatic equation (3.10) to the time-dependent equation (3.11) is found by considering figure 2. A current density flows in wires to charge capacitor plates and produces a changing ***E***-field between the plates. Using Stoke's theorem, one can rewrite (3.10) in terms of the line integral of the magnetic field around the path that bounds surface *A* and the surface integral across surface *A* where
3.12
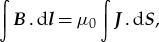

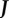
 is the current density in the wire and 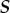 is the cross-sectional area of the wire. However, for (3.12) to describe the 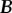-field produced in figure 2 correctly, the line integral 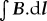 must not depend on whether we choose surface *A* or surface *B* over which to complete the surface integral. The right-hand side of (3.12) is 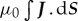 for surface *A* and zero for surface *B* because no current passes through surface *B*. To ensure that the line integral of 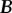 does not depend on whether surface *A* or surface *B* is chosen, and noting that the current density in the wire is given by 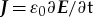, where 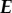 is the field between the plates, one can add Maxwell's displacement current density term to (3.10) to produce (3.11). Maxwell's brilliant addition led to the unification of electricity and magnetism.

#### (ii) Maxwell's third equation

In deriving Maxwell's third (and fourth) equation, we assume the system is constrained by the conservation of charge. The constraint implies
3.13
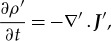

where 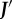 and 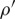 are the current density and charge density at the point 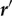. We have used the standard definition (cf equation (3.3))
3.14
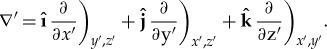


For this operator, similarly to equation (3.3), in addition to the primed spatial variables explicitly shown, the variable *t* and the non-primed variables *x*, *y* and *z*, are also held constant. Substituting (3.13) into (3.2) and then using standard vector field manipulations that include changing the order of partial derivatives and the vector field identity 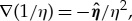 we find that
3.15
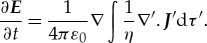


Textbook vector field algebraic techniques used in magnetostatics for functions of just three spatial variables can be used to rearrange the right-hand side of (3.15). Using the identity
3.16
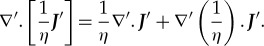

and then integrating gives
3.17
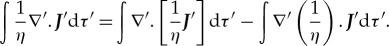


The second of the three integrals in (3.17) can be written as a surface integral and then set to zero, because without loss of generality, we can assume 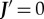 over the surface that defines 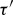. Using (3.17), 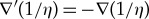, (3.16) with the del operator unprimed and 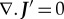 (because ∇ is not primed but 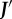 is primed) (3.15) then becomes:
3.18
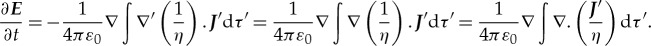


Using the vector field identity for the curl of the curl of a vector field, 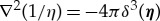, the vector field identity for the curl of the product of a vector field and a scalar, 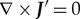, and 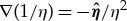 gives
3.19
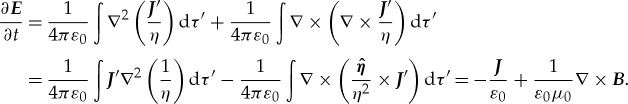


Hence, we find the third of Maxwell's equations:
3.20
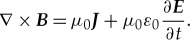


We can compare the mathematical approach we have used to derive (3.20) to Maxwell's heuristic approach. Maxwell considered a steady-state system, whereas this paper considers a local system. Both have invoked Occam's razor to generalize Coulomb's law of electrostatics to find an expression for 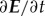 and are sufficiently general to find the same underlying differential equation (3.20).

### (d) The curl of ***E***

#### (i) Faraday's Law

Textbooks show that Coulomb's law for electrostatics [7] leads to
3.21
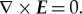


**Figure 3.**
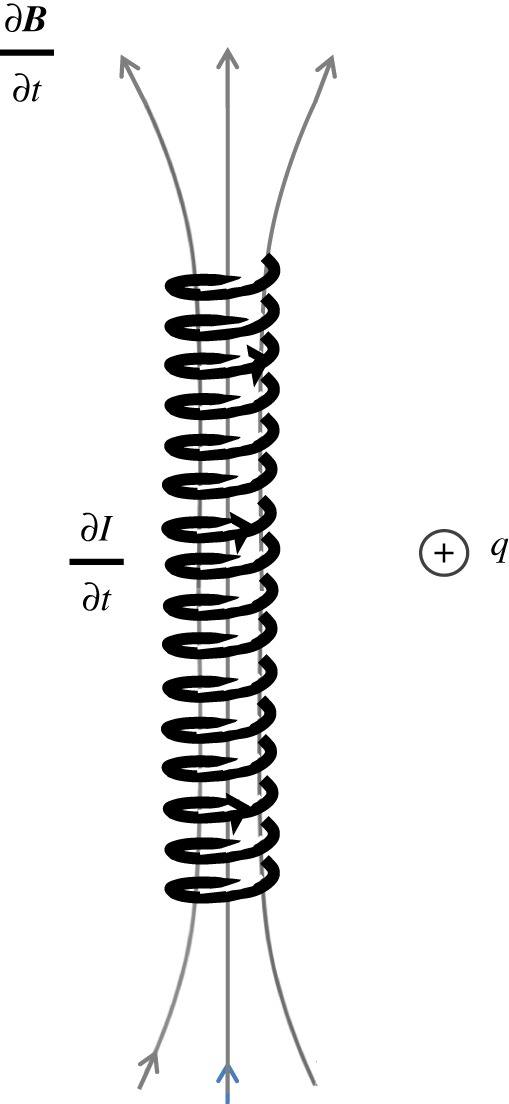
A stationary positive charge (*q*) outside a long solenoid. The current flowing in the solenoid is being increased (i.e. 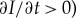
as is the magnetic field inside the solenoid (i.e. 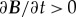). (Online version in colour.)

Equations (3.5), (3.9), (3.10) and (3.21) in time-independent form are known as the equations of electrostatics and magnetostatics. The Helmholtz theorem tells us that a vector field is completely specified by knowing its divergence and curl [12]. To generalize (3.21) to include time dependence, Maxwell used Faraday's experimental results [13]. Faraday found that if the magnetic field is steadily increased inside a long solenoid, there is a force on a stationary charge outside the solenoid (cf. figure 3). He measured the force on such stationary charges using loops of metallic wires (carrying unbound stationary charges) attached to voltmeters. Faraday's experiments (together with Lenz's experiments [14]) can be described mathematically as
3.22
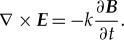

where *k* is a constant of proportionality approximately equal to unity. In order to constrain *k* to be exactly unity, some textbooks then assume that (3.22) is invariant under a Galilean transformation and then further assume this result remains true even for systems where relativistic effects are important. Such assumptions are not employed in the derivation of Faraday's Law below.

#### (ii) Maxwell's fourth equation

We first consider the primed partial time derivative of equation (3.20) which is
3.23
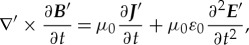

where 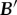, 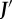 and 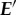 are the magnetic field, the current density and the 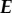-field at the position 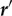. Substituting for 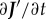 in (3.7) and using the vector field identity
3.24
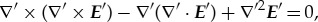

gives
3.25
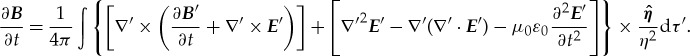


Using the vector field identity
3.26
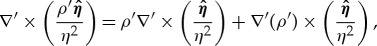

and given the curl of a radial function is zero, the second term in (3.26) is zero. Hence, using the vector field identity 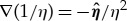 and (3.26), we can rewrite the second term in the second square bracket of (3.25) as
3.27
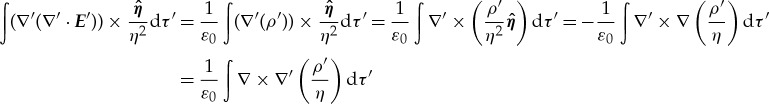


Using the vector field identities 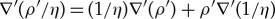 and 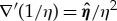 gives
3.28
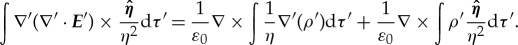


In using Coulomb's Law and Ampère's Law, we have ignored the internal structure of any element of charge density and current density. Hence without loss of generality, we assume that the volume occupied by every element of charge density and current density can be considered negligible and set the second integral in (3.28) to be zero. Therefore
3.29
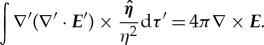


Substituting (3.29) into (3.25) then gives
3.30
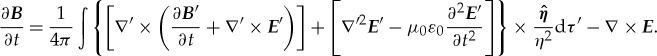


Satisfying (3.30) (together with the other Maxwell equations (3.5) and (3.24) already derived) is equivalent to requiring
3.31
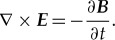


Equation (3.31) equates the left-hand side of (3.30) with the last term on the right-hand side. The primed version of (3.31) sets the first square bracket to zero. Given we already have Maxwell's equations (3.5) and (3.24), we can take them in primed form together with the primed version of (3.31) and the mathematical identity (3.24) to derive the wave-equation for 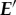 propagating through vacuum (i.e. 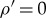 and 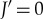) which is of the form 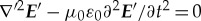. This ensures the second square bracket in (3.30) is zero. Hence, given Maxwell's first three equations, (3.31) is a solution to (3.30). We note that an alternative solution to (3.30) is of the form of (3.21). In the context of this paper, Maxwell's first three equations together with equation (3.21) provide an alternative set of four time-dependent differential equations for electromagnetism. We put this set of equations aside as non-physical, because they imply that any change in charge density or current density would instantaneously change the ***E***-fields and ***B***-fields throughout the entire Universe. Equation (3.31) is Faraday's Law. It is the fourth of Maxwell's equations. We have shown that Coulomb's Law, Ampère's Law and the conservation of charge are sufficient to expect Faraday's Law and that the value for the constant *k* in equation (3.22) is not a matter for experimentation but is fixed to be unity. Faraday's Law can be derived without any relativistic assumptions about Lorentz invariance.

## 4. Are Maxwell's equations universally true?

The Jefimenko equations [15] are the general solutions to Maxwell's four equations where
4.1
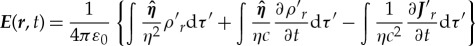

and
4.2
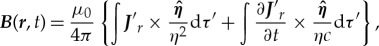

given that 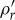 and 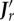 are subject to the constraint of the conservation of charge (cf. equation (3.13)). In the Jefimenko equations, the charge density, 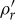, and current density, 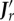, are calculated at the retarded time 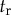 where 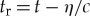. Jefimenko pointed out that his equations show that the fields are caused by the charge densities and current densities in the system and that when Maxwell added the displacement current density to his fourth equation, he coupled 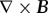 and 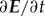 but did not introduce ‘a cause and effect relationship’ [15]. Similarly, this paper shows that Faraday's Law (in the differential form given by equation (3.31)) couples 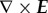 and 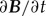 as a result of the conservation of charge, but they also should not be considered to be in ‘a cause and effect relationship’. It is interesting to identify those terms in the Jefimenko general solutions that were used by Maxwell and those used in this paper to help identify Maxwell's four underlying differential equations. Maxwell used Coulomb's Law and Faraday's Law associated with just two of the three terms for 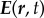 in equation (4.1). By also turning to Ampère's Law and the conservation of charge, he identified both terms for 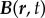 in equation (4.2). He avoided the complexities of retarded time by considering a steady-state system. In this paper, the central assumptions that Coulomb's electrostatic static law and Ampère's magnetostatic law are both true in the extreme local limit as a function of time is confirmed by Jefimenko's equations (i.e. the leading terms in equations (4.1) and (4.2) are equations (3.1) and (3.6), respectively). The complexity of retarded time is avoided by considering a local system where 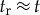. The ***E***-fields and ***B***-fields are coupled by the conservation of charge.

The experimental evidence for Maxwell's equations is overwhelming. Furthermore, as the gateway to Einstein's theory of relativity [16], which in itself also brings its own compelling experimental evidence, any speculation about charge creation or the breakdown of Maxwell's equations, is very probably destined to be fruitless. However, because we have derived Maxwell's equations using the conservation of charge as a constraint, we complete the paper by considering what would happen if this constraint did not always apply, or more precisely, where might we look for the breakdown of Maxwell's equations. We suggest that the essence of an entity that has been *created* is that there should be no experimental methods that can determine the properties of the created entity prior to creation. The probability of the entity's existence can be considered to increase from zero to one. Looking for events described by this language of probability naturally points us towards quantum mechanics. Given that quantum mechanics has been tested to exquisite accuracy and that all known interactions conserve charge, it becomes a remote possibility at best, that we can find charge creation. Alternative tests of Maxwell's equations include looking for the creation of current density, or electric and magnetic waves that do not obey Maxwell's equations. Our best chances are to seek out events that are so difficult to produce that they have not been extensively interrogated experimentally, and hence may offer something completely unexpected. We suggest investigating an Einstein–Podolsky–Rosen experiment [17]. Typically, an entangled electron–positron pair is mixed and prepared as a superposition of states with equal and opposite magnetic moments (or spins). The charges are separated and the magnetic moment or spin of the electron is measured. The well-known instantaneous collapse of the wavefunction occurs, so that the positron ends up with the opposite magnetic moment (or spin) to the electron. The appearance of the moment of the positron is triggered by entirely quantum mechanical effects—no direct electromagnetic communication occurs between the electron and positron. Indeed, one can think about the two charges as a single entity. However, one can argue that we do not really know how the information that leads to the positron producing a magnetic moment of opposite sign is instantaneously received—beyond asserting it is part of the fabric of quantum mechanics, or part of the nature of a macroscopic wavefunction. We suggest that while the moment of the positron is being *created* (rather than excited), the production of the ***B***-field associated with its magnetic moment may not be coupled to the production of any ***E***-field at all. So, one could measure 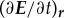 and 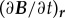 in the wavefront of the positron, hoping to find ***B***-fields with ***E***-fields that are inconsistent with Maxwell's equations.

## 5. Conclusion and final comments

Although Euclid's choices of starting points or axioms for geometrical mathematics seem obvious, Feynman has reminded us that they are not a unique set. We can use Euclid's axioms to derive Pythagoras' theorem or we can take Pythagoras' theorem as an axiom and drop one of Euclid's axioms [18]. Hence, we have the paradox that although Euclid's choices have remained generally accepted for centuries because they are closest to being self-evident truths, none of them is indispensable. The choice of axioms ultimately includes some of the ‘beauty is truth, truth beauty’ [19] sentiment. At the moment, most of the scientific community uses Feynman's seven equations of classical physics, including Maxwell's equations, as axioms. If we discover charge creation, or electric and magnetic waves that do not obey Maxwell's equations, then treating Maxwell's equations as axioms would become untenable. In this paper, we have shown that Maxwell's equations can be justified using a mathematical derivation that follows from Coulomb's Law, Ampère's Law and the conservation of charge. Therefore, as with other differential equations in physics, in the unlikely event that Maxwell's equations are not true under all circumstances, we can discuss how the equations are derived and make other choices of axioms.

## Data accessibility

The data and figures in the paper are available at: http://dx.doi.org/10.15128/gh93gz492 and associated materials are on the Durham Research Online website: http://dro.dur.ac.uk/26207/.
